# Machine learning prediction of pathologic myopia using tomographic elevation of the posterior sclera

**DOI:** 10.1038/s41598-021-85699-0

**Published:** 2021-03-26

**Authors:** Yong Chan Kim, Dong Jin Chang, So Jin Park, In Young Choi, Ye Seul Gong, Hyun-Ah Kim, Hyung Bin Hwang, Kyung In Jung, Hae-young Lopilly Park, Chan Kee Park, Kui Dong Kang

**Affiliations:** 1 Department of Ophthalmology, Incheon St. Mary’s Hospital, College of Medicine, The Catholic University of Korea, Seoul, Republic of Korea; 2 Department of Medical Informatics, College of Medicine, The Catholic University of Korea, Seoul, Republic of Korea; 3 Department of Ophthalmology, Yeouido St. Mary’s Hospital, College of Medicine, The Catholic University of Korea, Seoul, Republic of Korea; 4 Department of Ophthalmology, Seoul St. Mary’s Hospital, College of Medicine, The Catholic University of Korea, Seoul, Republic of Korea; 5 Department of Ophthalmology, Incheon St. Mary’s Hospital, College of Medicine, The Catholic University of Korea, 56, Dongsu-ro, Bupyeong-gu, Incheon, 21431 Republic of Korea

**Keywords:** Biomarkers, Medical research

## Abstract

Qualitative analysis of fundus photographs enables straightforward pattern recognition of advanced pathologic myopia. However, it has limitations in defining the classification of the degree or extent of early disease, such that it may be biased by subjective interpretation. In this study, we used the fovea, optic disc, and deepest point of the eye (DPE) as the three major markers (i.e., key indicators) of the posterior globe to quantify the relative tomographic elevation of the posterior sclera (TEPS). Using this quantitative index from eyes of 860 myopic patients, support vector machine based machine learning classifier predicted pathologic myopia an AUROC of 0.828, with 77.5% sensitivity and 88.07% specificity. Axial length and choroidal thickness, the existing quantitative indicator of pathologic myopia only reached an AUROC of 0.758, with 75.0% sensitivity and 76.61% specificity. When all six indices were applied (four TEPS, AxL, and SCT), the discriminative ability of the SVM model was excellent, demonstrating an AUROC of 0.868, with 80.0% sensitivity and 93.58% specificity. Our model provides an accurate modality for identification of patients with pathologic myopia and may help prioritize these patients for further treatment.

## Introduction

Complications from pathologic myopia are a major cause of visual impairment worldwide^1–4^. Eyes with pathologic myopia may develop ocular pathologies in the macula, peripheral retina, and optic nerve^5,6^. Excessive anteroposterior elongation of the globe may induce posterior staphyloma and other associated retinochoroidal lesions that are presumably important factors in the development of these degenerative changes^7^. However, there is some confusion regarding the definition of the disease, possibly due to the lack of a quantitative explanation. The phrase “myopic maculopathy” represents a similar concept that also does not have an exact definition. In 1970, Curtin proposed a definition of myopic maculopathy that included the features of chorioretinal atrophy, Fuchs spot, lacquer cracks, posterior staphyloma, and optic disc changes^8^; however, thus far, there are no quantitative specifications of this disease that fully describe the condition of the posterior globe.

Since the introduction of fundus photography, many methods have been suggested for differentiation of pathologic myopic eyes. Recently, a photographic classification system and optical coherence tomographic criteria have been proposed^9,10^. Another classification system based on atrophy, traction, and neovascularization has been introduced using a similar approach^11^. Qualitative analysis of fundus photographs enables straightforward pattern recognition of advanced pathologic myopia; however, it has limitations in defining the classification of the degree or extent of early disease, such that it may be biased by subjective interpretation. Spectacle correction of the eye (in a measurement unit known as diopters) and axial length (AxL) are often used as quantitative indicators of pathologic myopia; however, these parameters, alone or in combination, do not accurately reflect the globe geometry for diagnosis of pathologic myopia^12,13^.

Our group recently proposed a novel method to represent geometrical information of the posterior globe by measuring the anteroposterior depth of routinely used optical coherence tomography (OCT) coronal scans^14,15^. This method uses the fovea, optic disc, and deepest point of the eye (DPE) as the three major markers (i.e., key indicators) of the posterior globe to quantify the relative tomographic elevation of the posterior sclera (TEPS). Using this quantitative index from eyes of 860 myopic patients, we investigated whether machine learning classifiers could discriminate the presence of pathologic myopia.

The present study aimed to propose an easy-to-use and clinically available model to identify patients with pathologic myopia, based on quantitative measurement of the posterior globe. We developed a kernel Support Vector Machine (SVM) prediction model using a large-scale myopic eye database, with features collected only by routinely used OCT apparatuses. The discriminative ability of our proposed model was compared with the abilities of conventional myopia indices, the AxL and subfoveal choroidal thickness (SCT). The discriminative ability of the proposed model was also compared with the abilities of other machine learning algorithms, namely Decision Tree, Random Forest, k-nearest neighbors, and Naïve Bayes.

## Material and methods

### Study population

**Figure 1:**
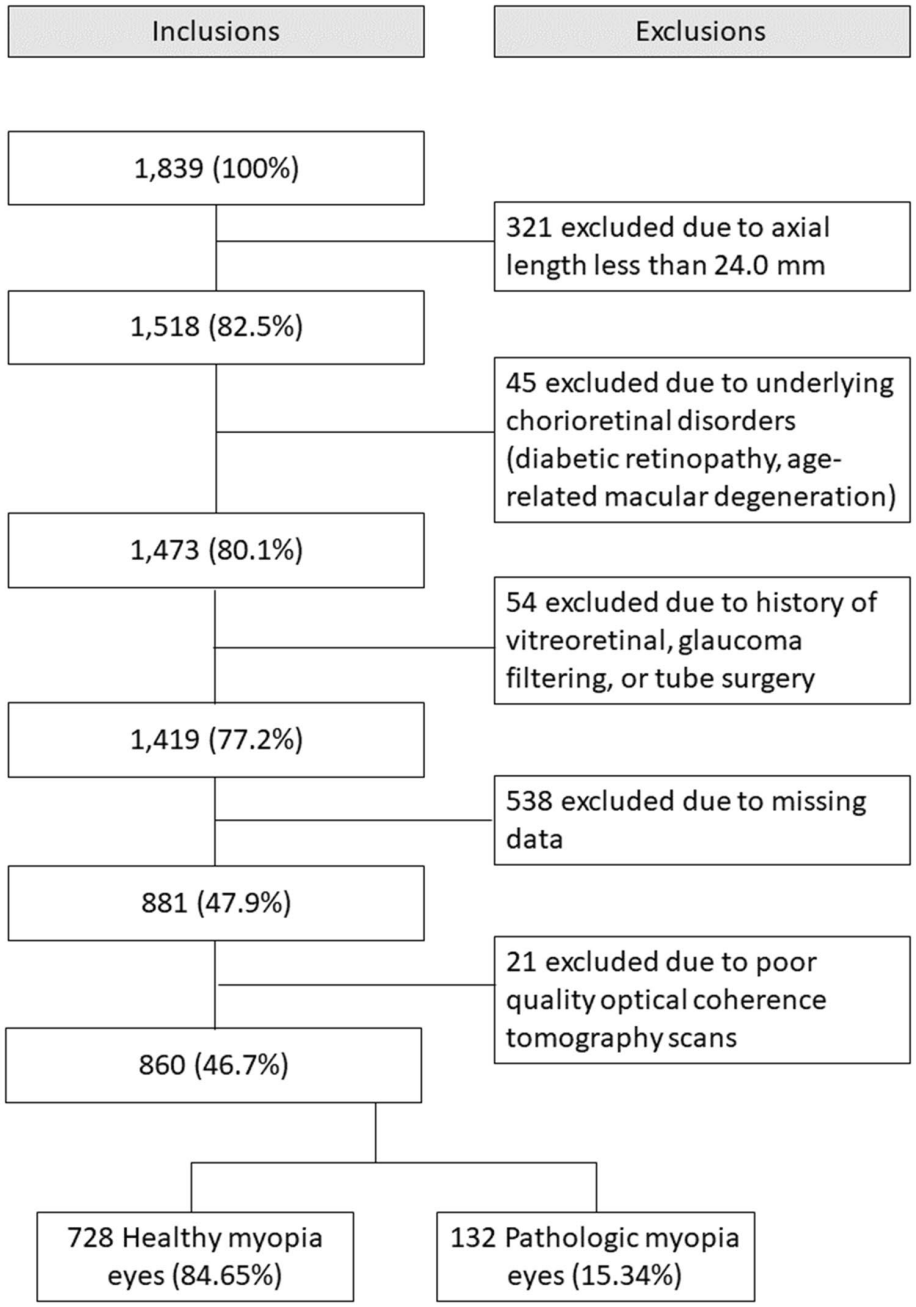
Inclusion, exclusion flowchart of the study participants.

This was a multicenter retrospective case series study. The data set was developed from information collected from 1839 patients who underwent clinical examinations in the ophthalmology clinics at Incheon Saint Mary’s Hospital (Incheon, Republic of Korea) and Seoul Saint Mary’s Hospital (Seoul, Republic of Korea), between January 2012 and May 2020. For data mining, patients who had any of the following conditions were excluded: AxL < 24.0 mm (n = 321); other retinal or choroidal disorders, such as diabetic retinopathy, retinal vascular diseases, or age-related macular degeneration (n = 45); poor quality OCT scans (n = 21); a history of vitreoretinal, glaucoma filtering, or tube surgery (n = 54); and missing data (n = 538) (Fig. 1). The study was conducted in accordance with the ethical standards stated in the 1964 Declaration of Helsinki and was approved by The Catholic University of Korea Institutional Review Board (IRB no. OC19RESI0161). Informed consent was obtained for each enrolled subjects.

The enrolled patients were divided into the following two groups: (1) healthy myopia patients with AxL > 24.0 mm and no pathological changes and (2) pathologic myopia patients with AxL > 24.0 mm and pathological myopic changes, determined in accordance with the guidelines specified by the International Myopia Institute^16^. The International Myopia Institute defines pathologic myopia as an excessive axial elongation associated with myopia that leads to structural changes in the posterior segment of the eye (including posterior staphyloma, myopic maculopathy, and high myopia-associated optic neuropathy) as well as a loss in best-corrected visual acuity^16^. Eyes with any type of posterior staphyloma and stages 2, 3, or 4 of the Meta-Analysis for Pathologic Myopia classification system, with or without “plus” lesions, were considered to have pathologic myopia in this study^9^. For reference, the Meta-Analysis for Pathologic Myopia system organizes myopic maculopathy into five stages: 0, no maculopathy; 1, tessellated fundus; 2, diffuse choroidal atrophy; 3, patchy chorioretinal atrophy; and 4, macular atrophy^17^. Plus lesions included three additional indicators: lacquer cracks, myopic choroidal neovascularization, and Fuchs spot^10^. Diffuse choroidal atrophy, determined using ophthalmoscopy, is an ill-defined yellowish lesion in the posterior fundus; patchy atrophy constitutes a grayish-white, well-defined atrophy; and lacquer cracks appear as fine, irregular, yellowish lines that often branch and crisscross in the fundus. Posterior staphyloma was defined and classified in accordance with the definition provided by Curtin and the International Myopia Institute: local bulging of the sclera at the posterior pole of the eye, with a radius less than the surrounding curvature of the eye wall^8,9,18,19^. Diagnosis and classification of posterior staphyloma using stereoscopic fundus photography was decided by agreement between two of the authors (YCK and KDK). The designation of healthy myopia or pathologic myopia was determined by two ophthalmologists (YCK and KDK). If the results from these two ophthalmologists were not consistent, a senior ophthalmologist (CKP) was consulted for the final judgment.

### Data collection and definition of variables

All patients underwent comprehensive clinical examinations, including refractive error (RE) in diopters, Landolt C chart best-corrected visual acuity measurements (measured using logarithm of the minimum angle of resolution), and slit-lamp biomicroscopy. AxL was measured using ocular biometry (IOL Master; Carl Zeiss Meditec, Jena, Germany). Digital color fundus photographs were taken with a VX-10i fundus camera (Kowa Co., Nagoya, Japan).

**Figure 2:**
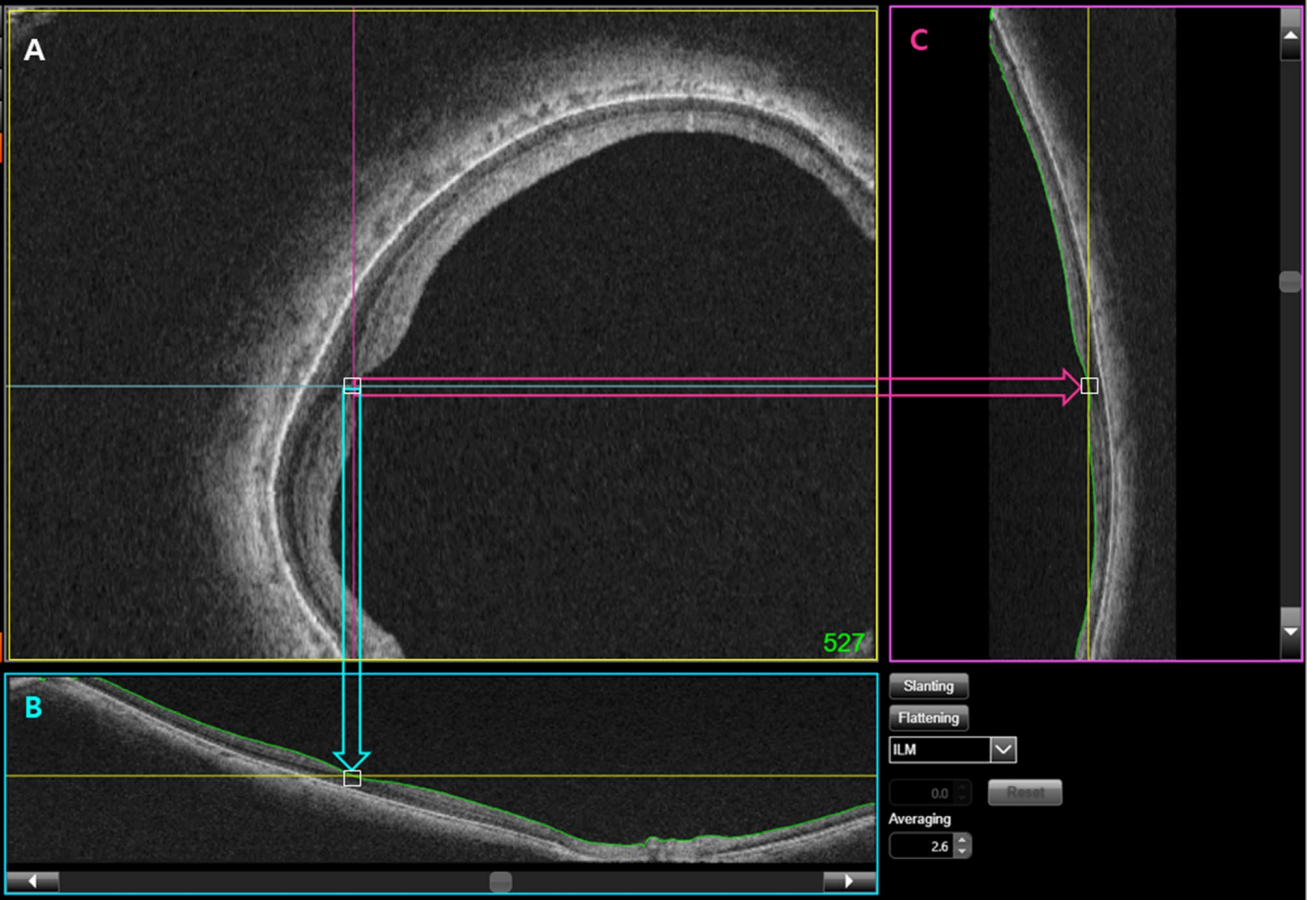
Designation of the fovea using the coronal (**A**), horizontal (**B**), and vertical (**C**) scan. The specific coronal section (given in green numbers in the (**A**)) that simultaneously displayed the foveal double hump in all three displays (**A**–**C**) was designated as the foveal position.

**Figure 3:**
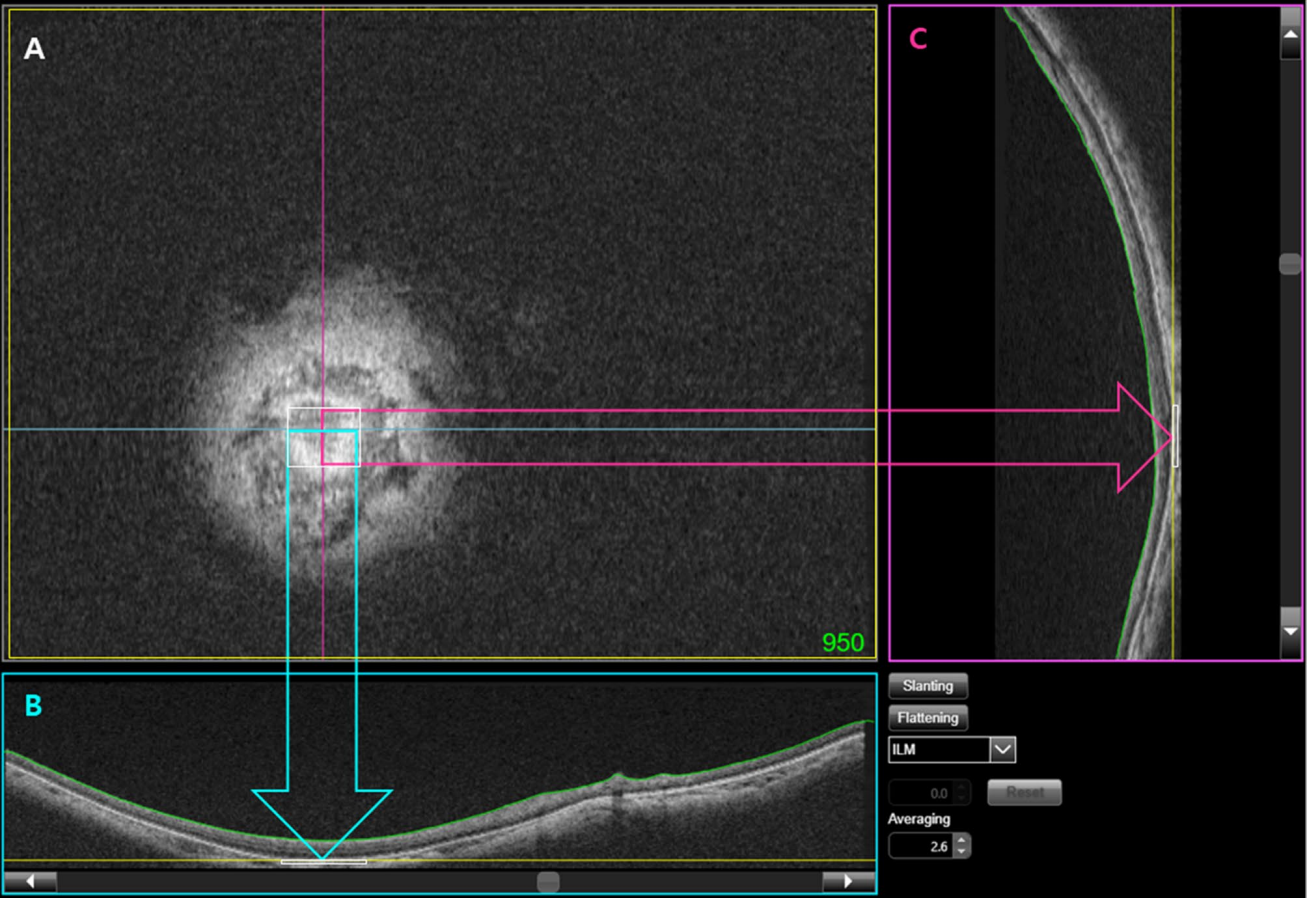
Designation of the deepest point of the eye (DPE) using the coronal (**A**), horizontal (**B**), and vertical (**C**) scan. The specific coronal section (given in green numbers in the (**A**)) that simultaneously displayed the coronal view of the hyperreflective Bruch’s membrane (white square) in all three displays (**A**–**C**) was designated as the DPE position.

**Figure 4:**
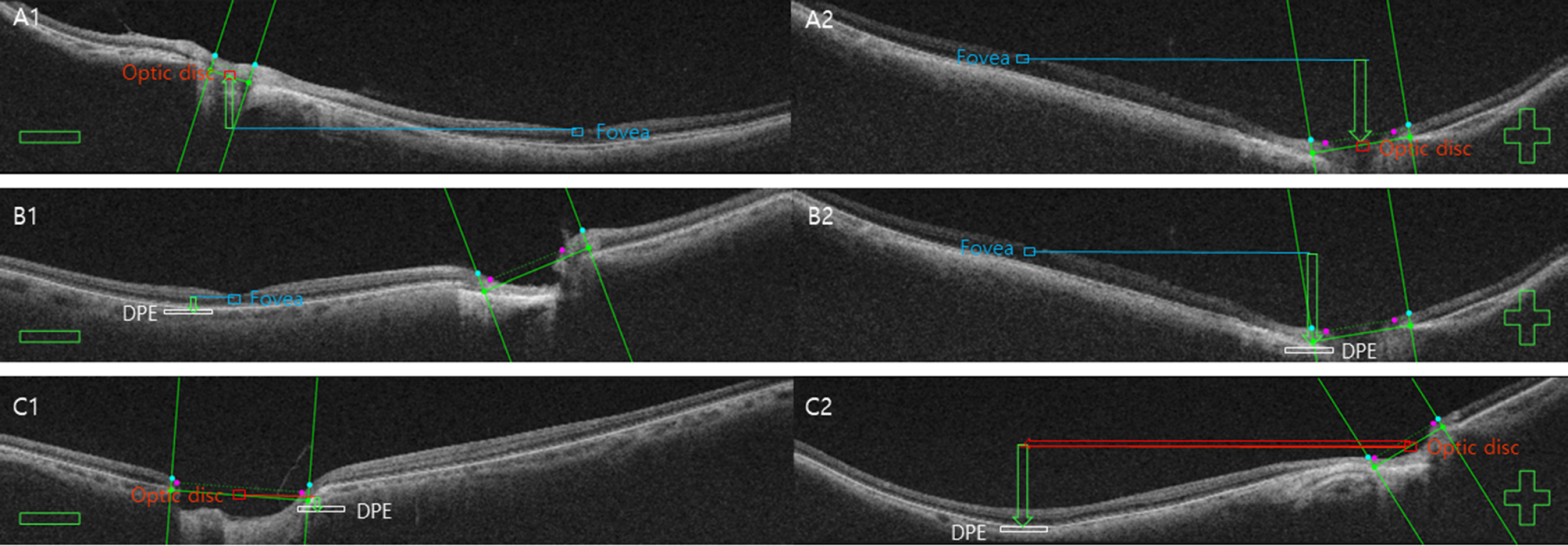
The concept of using relative anteroposterior elevation of the posterior globe between the fovea (blue square), the DPE (white square), and the optic disc (red square). The tomographic elevation from the fovea to the optic disc center (disc) was designated TEPS_fovea→disc_ (**A**); from the fovea to the DPE, TEPS_fovea→DPE_ (**B**); from the disc to the DPE, TEPS_disc→DPE_ (**C**); and perpendicular distance from the disc to the DPE, TEPS_distance_ (red arrow in (**C2**)). The tomographic elevation was estimated as the number of coronal sections between key indicators, with adjacent sections separated by 2.6 μm. The direction to the posterior (optic disc side) was specified as a positive tomographic elevation (**A2**,**B2**,**C2**). The direction opposite from the anterior (corneal side) was designated as the negative tomographic elevation (**A1**,**B1**,**C1**).

All enrolled patients were imaged by OCT (DRIOCT Triton; Topcon Corporation, Tokyo, Japan). The scanning protocol consisted of 256 B-scans centered on the fovea, which provided an image of the posterior segment 12 mm horizontally and 9 mm vertically. In total, 1000 consecutive coronal scan images were reconstructed, each with a separation of 2.6 μm. A good set of scans with a signal quality index of > 75 in the B-scan mode was selected for further analysis. Each key indicator section of the posterior sclera (i.e., (1) the fovea, (2) the DPE, and (3) the optic disc) was designated and documented as follows. The reviewers examined the reconstructed consecutive coronal scans (en face mode) from front (corneal side) to back (optic nerve side). (1) The coronal scan (Fig. 2A), horizontal scan (Fig. 2B), and vertical scan (Fig. 2C) were reviewed simultaneously; the specific coronal section (given in green numbers in the A section) that simultaneously displayed the foveal double hump in all three displays (Fig. 2A–C) was designated as the foveal position. (2) In a similar manner, the coronal scan (Fig. 2A), horizontal scan (Fig. 3B), and vertical scan (Fig. 3C) were reviewed simultaneously; the specific coronal section (given in green numbers) that simultaneously displayed the coronal view of the hyperreflective Bruch’s membrane (white square) in all three displays (Fig. 3A–C) was designated as the DPE position. (3) The optic disc position was measured by means of the automatic segmentation algorithm of DRIOCT software (Topcon Corp.) using Bruch’s membrane opening (green parallel lines in Fig. 4). The center of the line connecting Bruch’s membrane opening (red square in Fig. 4) at the optic disc center was designated as the optic disc center position. In the extreme cases where the inferior margin of DPE cannot be determined, we assumed that the DPE is within the inferior boundary of the scanning field. Eyes with segmentation errors involving Bruch’s membrane opening were manually measured by two of the authors; the final position was designated by agreement.

The relative elevation in the posterior globe was estimated by assignment of a specific anteroposterior position for each of the three key indicators, using four indices collectively referred to as TEPS. Specifically, the four indices were as follows: (1) the tomographic elevation from the fovea to the optic disc center (disc) was designated TEPS_fovea→disc_ (Fig. 4A); (2) from the fovea to the DPE, TEPS_fovea→DPE_ (Fig. 4B); (3) from the disc to the DPE, TEPS_disc→DPE_ (Fig. 4C); and (4) perpendicular distance from the disc to the DPE, TEPS_distance_ (red arrow in Fig. 4C2). The tomographic elevation was estimated as the number of coronal sections between key indicators, with adjacent sections separated by 2.6 μm^14^. The direction to the posterior (optic disc side) was specified as a positive tomographic elevation (Fig. 4A2,B2,C2). The direction opposite from the anterior (corneal side) was designated as the negative tomographic elevation (Fig. 4A1,B1,C1). The TEPS perpendicular distance was estimated by measuring the linear distances in micrometers in the 3D reconstructed configuration, which considers the extreme diagonal disc, DPE configuration of the posterior sclera, between key indicators using the intrinsic calipers in DRIOCT software. The SCT was defined as the perpendicular distance from the outer edge of the hyperreflective line of Bruch’s membrane to the choroidoscleral junction at the subfovea. Measurements from vertical and horizontal B-scan images, including the fovea, were averaged. All reviews of the instrumentation were carried out by one masked author (YCK); the 1:1 pixel mode was converted to the 1:1 µm mode for greater accuracy^15^.

To compensate for potential scanning errors induced by head tilt or ocular rotation, the examiner confirmed the patient’s position at the OCT with their chin in the chin rest and forehead against the forehead rest. The patient’s eyes were aligned with the eye level mark on the forehead rest support by raising or lowering the chin rest. For determination of the foveal center, patients were instructed to hold their heads in a vertical position and look directly at the internal fixation target in the OCT camera. The OCT apparatus also was equipped with real-time eye tracking to eliminate eye motion and minimize artifacts by fixation on the fovea for each scan^14^.

To evaluate measurement repeatability, two separate scan sets were collected from 14 eyes from each group; the topographic locations of the three key indicators were compared. The intraclass correlation coefficient and coefficient of repeatability were calculated. The coefficient of repeatability is defined as the standard deviation of the difference between two sets of scanned image measurements, divided by the average of the two repeated measurements^20^. Bland–Altman plots were also used to assess agreement between the two repeated measurements (Supplementary Fig. S1)^21^.

### Machine learning algorithm construction

In this study, an SVM-based classification algorithm was used to build classification models based on the different combinations of variables described above. Decision Tree, Random Forest, k-nearest neighbors, and Naïve Bayes classifiers were used to develop a prediction model based on the combination of the aforementioned variables.

The data were divided into a training set (70%) and a test set (30%) to obtain a reliable evaluation and to avoid overfitting. In the training set, the class imbalance ratio was 84.7 to 15.3. To resolve the imbalance, the Synthetic Minority over-sampling TEchnique (SMOTE) was used to identify an individual in the low-portion group and find its k-nearest neighbor, thereby creating a new data set for the low-portion group; k was set at 5 in this particular model. After SMOTE, the training data consisted of 970 eyes: 510 healthy eyes (52.6%) and 460 pathologic myopic eyes (47.4%). The model was then retrained with these data to construct the final prediction model. Using the final training model, independent validations were performed on the original data.

Based on the steps described above, the specific machine learning algorithms were trained as follows. First, individual pairs consisting of two of the four TEPS indices were selected to create SVM classifier models. Second, an SVM model using all four TEPS indices was constructed. Third, AxL and SCT were used to build an SVM model. Fourth, all six indices were used to develop an SVM model. Finally, SVM, Decision Tree, Random Forest, k-nearest neighbors, and Naïve Bayes classifiers were constructed using all six indices.

The machine learning algorithm was implemented using R version 3.6.2 (R Foundation for Statistical Computing). The predictive performance was compared by calculating (1) accuracy, which constitutes the overall correctness of the model (i.e., number of correct classifications divided by total number of classifications); (2) sensitivity, which evaluates a model’s ability to predict the true positives of each available category; (3) specificity, which assesses a model’s ability to predict the true negatives of each available category; and (4) area under the receiver operating characteristic curve (AUROC).

### Support vector machine architecture

An alternative use for SVM is the kernel method, which enables the modeling of higher dimensional, nonlinear models^22^. In a nonlinear problem, a kernel function can be used to add new dimensions to the raw data, thus converting the nonlinear problem into a linear problem in the resulting higher dimensional space. Briefly, a kernel function facilitates more rapid calculations, which would otherwise require computations in high-dimensional space^23^. With kernel functions, the scalar product between two data points in a higher dimensional space can be calculated without explicit calculation of the mapping from the input space to the higher dimensional space^23^. For our model, the most commonly used radial basis function (RBF) kernel is applied, in which the corresponding feature vector is infinite-dimensional:

$$\mathrm{K}\left({x}_{i},{x}_{j}\right)=\mathrm{exp}\left(-\gamma {\Vert {x}_{i}-{x}_{j}\Vert }^{2}\right).$$

Here, $\gamma$ is associated with the Gaussian function standard deviation, in which $\gamma$ size is related to overfitting. To find the optimal parameters, the training of kernel SVM was performed on the model 11. The best parameter for gamma was 1 and 10 for cost. The second best parameter for gamma and cost was 1, respectively (Supplementary Table S1). We compared the performance of each of the two best models and the default parameters (gamma = 1/data dimension, Cost = 1) (Supplementary Table S2). The model that showed the best performance was the model with 1/data dimension for gamma and 1 for cost.

### Statistical analysis

Continuous variables are presented as the mean ± standard deviation, while categorical variables are presented as frequencies and percentages. Differences between groups were analyzed using Fisher’s exact test for categorical variables and Welch’s t-test (or the Wilcoxon rank-sum test) for continuous variables. Statistical analyses were performed using R version 3.6.2 (R Foundation for Statistical Computing). *P-*values < 0.05 were considered statistically significant.

## Results

The demographics and clinical features of the patients in this study are listed in Table 1. The mean age of the total cohort was 52.43 ± 14.14 years; 59.3% of the patients were men. The mean AxL, RE, and best-corrected visual acuity were 26.00 ± 1.61 mm, − 4.62 ± 3.43 diopters, and 0.09 ± 0.16 logarithm of the minimum angle of resolution, respectively. The number of eyes with posterior staphyloma was 105 (12.21%) and the number of eyes with worse than category 2 myopic maculopathy, with or without plus lesions, was 42 (3.19%); among these 42 eyes, 15 had coexisting pathologic myopia features. Thus, 132 eyes showed pathologic myopia. The remaining 728 eyes showed no pathologic myopia features. Compared with patients without pathologic myopia, patients with pathologic myopia were significantly older (*P* = 0.004), had significantly longer AxL (*P* < 0.001), and had significantly worse best-corrected visual acuity (*P* < 0.001; Table 1).

**Table 1:** Comparison of demographics and clinical features between patients with and without pathologic myopia.

Variables	Overall	Healthy myopia eyes	Pathologic myopia eyes	*P* value^†^
No. of eyes (%)	860 (100)	728 (84.65)	132 (15.34)	
Age (years old)	52.43 ± 14.14	51.80 ± 13.83	55.89 ± 15.31	**0.004**
Male, n (%)	510 (59.30)	451 (61.95)	59 (44.70)	** < 0.001**^**‡**^
Axial length (mm)	26.00 ± 1.61	25.68 ± 1.23	27.75 ± 2.25	** < 0.001**
BCVA (logMAR units)	0.09 ± 0.16	0.06 ± 0.11	0.22 ± 0.28	** < 0.001**
**Classification of staphyloma, n (%)**				
Type 1, n (%)	16 (1.86)	0	16 (1.86)	
Type 2, n (%)	26 (3.02)	0	26 (3.02)	
Type 3, n (%)	25 (2.91)	0	25 (2.91)	
Type 4, n (%)	6 (0.70)	0	6 (0.70)	
Type 5, n (%)	25 (2.91)	0	25 (2.91)	
Others, n (%)	7 (0.81)	0	7 (0.81)	
**Modified myopic maculopathy according to META-PM study, n (%)**				
Category 0 (no maculopathy)	687 (79.88)	663 (91.07)	24 (18.18)	
Category 1 (tessellated fundus)	131 (15.23)	65 (8.93)	66 (50.0)	
Category 2 (diffuse atrophy)	28 (3.26)	0	28 (21.21)	
Category 3 (patchy atrophy)	8 (0.93)	0	8 (6.06)	
Category 4 (macular atrophy)	6 (0.70)	0	6 (4.55)	
Myopic CNV	23 (2.67)	0	23 (17.42)	
Lacquer cracks	17 (1.97)	0	17 (12.89)	

*BCVA* best corrected visual acuity, *CNV* choroidal neovascularization, *No.* number.

Data are presented as mean ± standard deviation unless otherwise indicated.

^†^Independent *t*-test for continuous variables.

^‡^χ^2^ test for categorical variables.

^§^statistically significant values (*P* < 0.05) are shown in bold.

### Comparison of six variables for each group

Table 2 compares the six ocular measurement indices, including the four TEPS indices, AxL, and SCT. Pathologic myopic eyes showed significant TEPS_fovea→DPE_, TEPS_disc→DPE_, and TEPS_distance_, compared with the individuals without pathologic myopia (*P* < 0.001, *P* < 0.001, and *P* = 0.002, respectively). The difference in TEPS_fovea→disc_ was not statistically significant between the two classes (*P* = 0.924); however, the group with pathologic myopia had a much larger standard deviation (628.17 in pathologic myopia eyes versus 200.64 in healthy eyes). The standard deviations of the pathologic myopia class were much greater in all four TEPS measurements (628.17, 325.12, 422.19, and 1730.64, respectively). The AxL and SCT readings of patients with pathologic myopia were significantly larger (both *P* < 0.001). The data from the six ocular instrumentation parameters were applied to the advanced machine learning classification model.

**Table 2:** Six ocular instrumentation input in five classification model*.

Variables	Overall (n = 860)	Healthy myopia eyes	Pathologic myopia eyes	*P* value^†^
TEPS _fovea→disc_, (μm)	258.30 ± 307.00	257.50 ± 200.64	262.74 ± 628.17	0.924
TEPS _fovea→DPE_ (μm)	393.25 ± 192.42	351.46 ± 115.36	623.72 ± 325.12	** < 0.001**^**‡**^
TEPS _disc→DPE_ (μm)	134.95 ± 226.99	93.96 ± 133.42	360.99 ± 422.19	** < 0.001**^**‡**^
TEPS _distance_ (μm)	3344.91 ± 1481.59	3270.49 ± 1420.51	3755.33 ± 1730.64	**0.002**^**‡**^
Axial length (mm)	26.00 ± 1.61	25.68 ± 1.23	27.75 ± 2.25	** < 0.001**^**‡**^
SCT (μm)	243.12 ± 106.61	256.28 ± 102.40	170.53 ± 100.31	** < 0.001**^**‡**^

*SCT* subfoveal choroidal thickness; *TEPS* tomographic elevation of the posterior sclera.

*Data are presented as mean ± standard deviation unless otherwise indicated.

^†^Independent *t*-test for continuous variables.

^‡^Statistically significant values (*P* < 0.05) are shown in bold.

### Machine learning classifier

**Figure 5:**
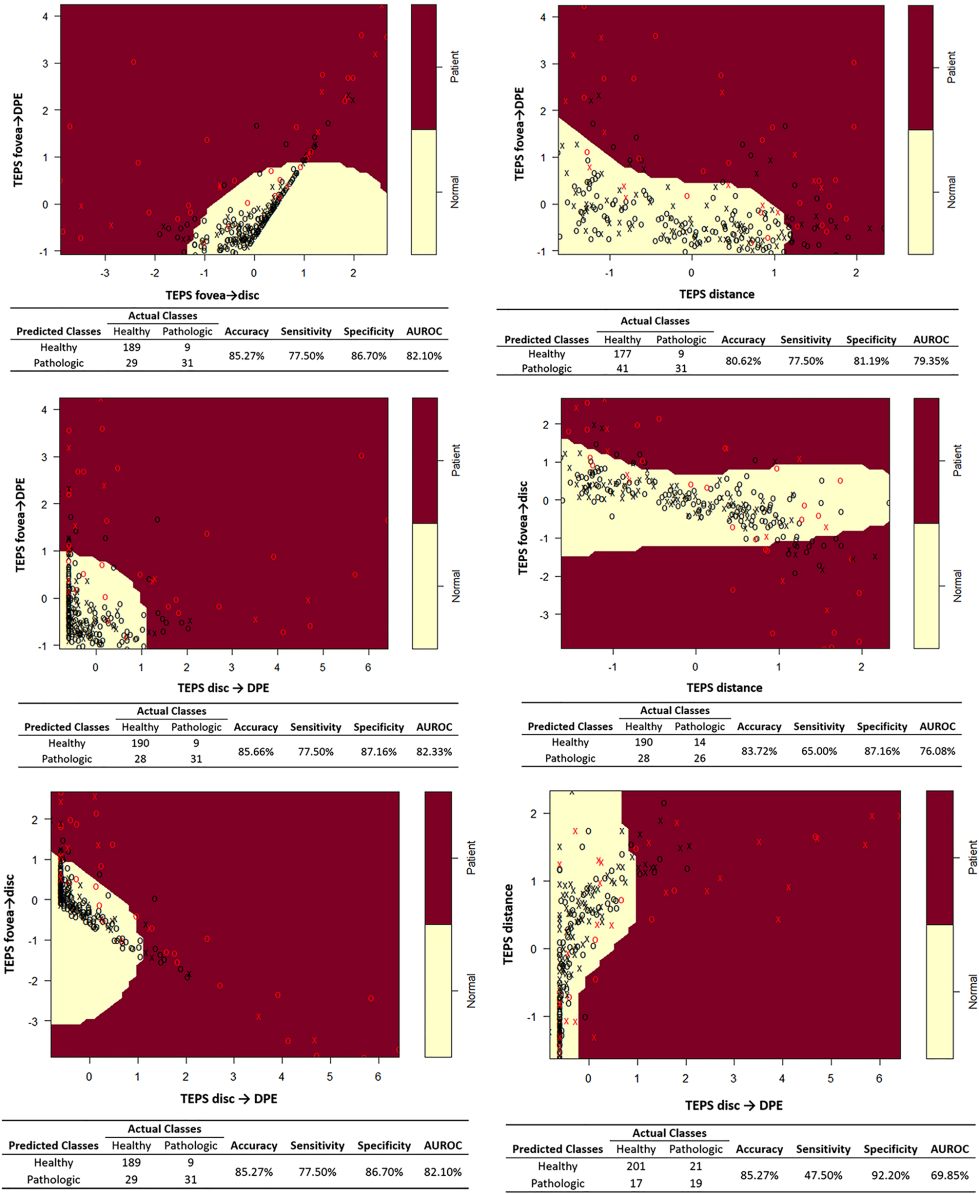
The two-dimensional plots and confusion matrix of individual pairs of the four TEPS indices. The nonlinear decision boundary classifying pathologic myopic eyes (red dot) and healthy eyes (black dot) is shown in each plot. Analysis was conducted in R (R Core Team 2014) and figures were produced using the package e1071^34^.

Table 3 shows the confusion matrix and the results of the SVM classifier, considering the 11 models constituting the six ocular measurements (four TEPS, AxL, and SCT). The models consisting of two of the four TEPS indices each showed at least better than 80.62% accuracy, 47.50% sensitivity, 81.19% specificity, and an AUROC value of 0.6985. Figure 5 show two-dimensional plots of individual pairs of the four TEPS indices. The nonlinear decision boundary classifying pathologic myopic eyes and healthy eyes is shown in each plot. The model using the two conventional indices (AxL and SCT) showed 76.36% accuracy, 75.0% sensitivity, 76.61% specificity, and an AUROC value of 0.7580. The model using all four TEPS indices demonstrated 86.43% accuracy, 77.50% sensitivity, 88.07% specificity, and an AUROC value of 0.8279; these values were better than those of the model using conventional indices. The model using all six measurements generated 90.31% accuracy, 82.50% sensitivity, 91.74% specificity, and an AUROC value of 0.8712, indicating excellent classification ability. Sensitivities were relatively low for all models, due to test set class imbalance.

**Table 3:** Confusion matrix (actual versus predicted classes), accuracy, sensitivity, specificity, AUROC of the support vector machine classification on each of 11 models.

Model	Variables	Predicted	Actual classes		Accuracy (%)	Sensitivity (%)	Specificity (%)	AUROC (%)
			Healthy	PM				
1	TEPS _fovea→disc_ and TEPS _fovea→DPE_	Healthy	189	9	85.27	77.50	86.70	82.10
		PM	29	31				
2	TEPS _distance_ and TEPS _fovea→DPE_	Healthy	177	9	80.62	77.50	81.19	79.3
		PM	41	31				
3	TEPS _disc→DPE_ and TEPS _fovea→DPE_	Healthy	190	9	85.6	77.50	87.16	82.33
		PM	28	31				
4	TEPS _distance_ and TEPS _fovea→disc_	Healthy	190	14	83.72	65.50	87.1	76.08
		PM	28	26				
5	TEPS _disc→DPE_ and TEPS _fovea→disc_	Healthy	189	9	85.27	77.50	86.70	82.10
		PM	29	31				
6	TEPS _distance_ and TEPS _disc→DPE_	Healthy	201	21	85.27	47.50	92.20	69.85
		PM	17	19				
7	4 TEPS variables	Healthy	192	9	86.43	77.50	88.07	82.79
		PM	26	31				
8	AL	Healthy	174	14	77.52	65.00	79.82	72.41
		PM	44	26				
9	CT	Healthy	165	15	73.64	62.50	75.69	69.09
		PM	53	25				
10	AL & CT	Healthy	167	10	76.36	75.00	76.61	75.80
		PM	51	30				
11	All variables	Healthy	204	8	91.47	80.00	93.58	86.47
		PM	14	32				

*AL* axial length, *AUROC* area under receiver operating characteristic curve, *CT* choroidal thickness, *DPE* deepest point of the eyeball, *PM* pathologic myopia, *TEPS* tomographic elevation of the posterior sclera.

**Figure 6:**
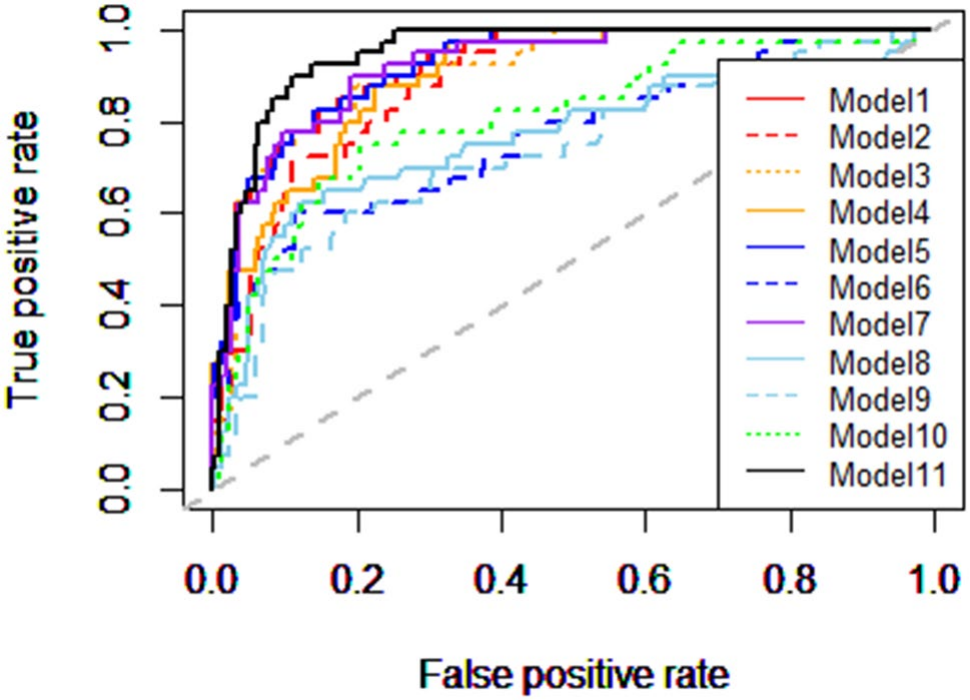
The AUROC values of each of the 11 models in Table 3. The Models 1–6 is the SVM applying two TEPS indices, the model 7 applied all 4 TEPS indices, the model 8–9 applied the conventional indices, and the model 11 used all 6 variables. Analysis was conducted in R (R Core Team 2014) and figures were produced using the package ROCR^35^.

Figure 6 shows the AUROC values of each of the 11 models. Two models with conventional indices (models 8 and 9) were comparatively incapable of classifying eyes with pathologic myopia. Models applying two TEPS indices (models 1–6) were mostly capable of distinguishing pathologic myopia, with the exception of model 6. The three models using the index TEPS_distance_ were comparatively incapable, compared with the other models (79.35%, 76.08, and 69.85% versus 82.10%, 82.33%, and 82.10%, respectively). The model using all four TEPS indices had no definitive advantage over models using two of the four TEPS indices (82.79% versus 82.10%). However, the model using all six measurement indices (model 11) showed 87.12% capability, which was superior to any other model.

**Figure 7:**
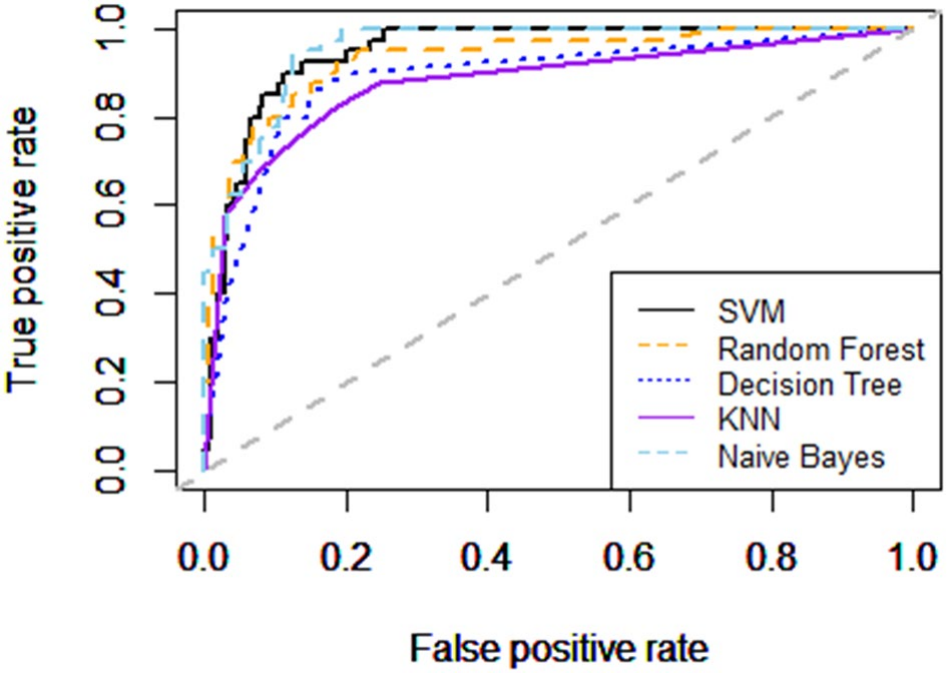
The AUROC values of five different machine learning models. Analysis was conducted in R (R Core Team 2014) and figures were produced using the package ROCR^35^.

Table 4 and Fig. 7 compares the classification results of the five machine learning algorithms using all six ocular instrumentation features. The RBF Kernel SVM machine learning algorithm showed the best sensitivity and AUROC, with accuracy and specificity similar to those of Decision Tree and Random Forest. Overall, the RBF Kernel SVM classifier maintained the best discriminative ability and balanced sensitivity and specificity.

**Table 4:** The performance of Kernel SVM, decision tree, random forest, KNN, and Naïve Bayes using all six ocular instrumentation features.

Model	Predicted	Actual classes		Accuracy (%)	Sensitivity (%)	Specificity (%)	AUROC (%)
		Healthy	PM				
Kernel SVM	Healthy	204	8	91.47	80.00	93.58	86.79
	PM	14	32				
Decision Tree	Healthy	186	8	84.50	80.00	85.32	82.66
	PM	32	32				
Random Forest	Healthy	204	10	90.70	75.00	93.58	84.29
	PM	14	30				
KNN	Healthy	190	10	85.27	75.00	87.16	81.08
	PM	28	30				
Naïve Bayes	Healthy	196	9	87.98	77.50	89.91	83.70
	PM	22	31				

*AUROC* area under receiver operating characteristic curve, *SVM* support vector machine, *PM* pathologic myopia.

## Discussion

In this study, we proposed an RBF Kernel SVM classifier using a posterior globe tomographic measurement-based data set to predict the presence of pathologic myopia in myopic eyes. Only six features were used in our model to produce 91.47% accuracy and an AUROC value of 0.865. Notably, the features of our model only required ocular OCT measurements. Clinical data such as the patient’s age, visual acuity, or any other ocular or systematic information were not needed. The novel TEPS measurement featured considerably superior discriminative ability, compared with the conventional measurement approach based on the AxL and SCT. Using all six ocular features (four TEPS, AxL, and SCT), the SVM classifier showed good discriminative ability and balanced sensitivity and specificity, compared with other machine learning algorithms. To the best of our knowledge, our classifier is the first attempt to automatically detect pathologic myopia with high accuracy.

Patients with high myopia require accurate diagnosis of whether pathologic myopia is present, because patients with this condition exhibit an increased risk of visual impairment. This task is a great challenge for the ophthalmologist, because no quantitative standards are available for the diagnosis of pathologic myopia. The current method for diagnosis of this pathologic condition involves comparison with standard photographs, along with clinical experience^9^. Qualitative analysis by subjective pattern recognition is substantially biased according to personal experience and data quality. This problem may be resolved by acquisition of specific measurements of the posterior pole and incorporation of these parameters into machine learning; this comprises the development of a novel algorithm that automatically and objectively classifies an eye within a defined category, based on a combination of various posterior scleral measurements.

This study showed that the application of SVM, a machine learning technique, to posterior scleral OCT measurements can be used to accurately classify whether myopic eyes exhibit healthy myopia or pathologic myopia. Analysis of conventional parameters alone is insufficient to discriminate between eyes with healthy myopia and eyes with pathologic myopia. Using specific tomographic data from the posterior globe increases the accuracy, sensitivity, specificity, and AUROC of the SVM classifier. This result is in good agreement with substantial evidence suggesting that changes to the shape of the globe are responsible for the macular, peripheral, and optic disc alterations described in these patients^5,17,24^. Table 3 shows that the use of the TEPS measurement is crucial in the detection of pathologic myopia; specifically, 86.43% of the included eyes were correctly classified when using the TEPS measure, compared with 76.36% when only conventional AxL and SCT were evaluated. When all measurements were considered, only 8.25% of healthy eyes were incorrectly presumed to exhibit pathologic myopia (Table 3).

A major advantage of our SVM prediction model is that it is based on measurable parameters that can easily be collected in ophthalmology clinics where OCT is available. Currently, clinics are likely to have OCT devices; many retinal examinations include OCT as a routine procedure. Whereas some methods require magnetic resonance imaging, which may be difficult to acquire and difficult to quantify, such features were not included in our model development^25,26^. Similarly, we presumed that single-office visual acuity and intraocular pressure measurements may not be fully representative of clinical conditions; therefore, these were not included as input features.

Various attempts have been made to quantify the geometry of the posterior sclera. Park et al.^24^ used staphyloma height, curvature index, and coefficient alpha to quantify the geometry of posterior sclera; they found that a steeper change in foveal curvature was related to myopic tractional maculopathy, but not myopic choroidal neovascularization. Akagi et al.^27^ measured the scleral bending angle at the peripapillary area and suggested that the amount of bending angle may be correlated with reduced retinal nerve fiber layer thickness. In contrast, our posterior scleral measurement is designed to evaluate the contour elevation of the posterior globe. TEPS represents the anteroposterior depth difference among the three posterior key indicators in the three-dimensional plot. Because all three indicators are contained within the three-dimensional contour of the posterior globe, each of the four TEPS indices must be highly correlated with the others. Thus, scatter plots constituting two of the four TEPS indices showed the dot distribution confined to a singular line. In Fig. 4, consecutive black dots (healthy eyes) assembled to form a main singular line in the yellow (normal) territory, whereas most of the red dots (eyes with pathologic myopia) were spread throughout various places in the red (patient) territory. Accordingly, eyes with smooth contours exhibited geometric measurements that were highly correlated with each other, forming a singular line (black dots). In contrast, eyes with uneven contours exhibited geometric measurements that were outliers from the main correlation line, which is consistent with the definition of posterior staphyloma. As described by Spaide, posterior staphyloma is an outpouching of a circumscribed posterior fundus region, which has a radius of curvature smaller than that of the adjacent eye wall^28^. In the two-dimensional TEPS plot, an outpouching of the posterior sclera constitutes an outlier from the line assembled from a smooth, physiologic eye. Our SVM classifier constructed an optimal nonlinear hyperplane on these plots as the decision surface between classes, which is an appropriate method to discriminate structural outliers of the posterior globe^29^. The polynomial kernel method of RBF SVM uses a nonlinear hyperplane to discriminate between classes, which is also appropriate for our data set characteristics^30^.

The AxL and RE are gold standards for representation of myopia; however, the SVM classifier using the AxL measurement does not have discriminative ability sufficient for use in clinical practice. According to our results, the use of AxL and RE for pathologic myopia discrimination presents a risk of missing pathologic eyes that may not have a long AxL or RE^31^. Regarding the SCT, Fang et al.^10^ reported that progressive choroidal thinning plays a major role in the progression of myopic maculopathy, followed by many other similar findings^32,33^. However, SCT alone did not have any discriminative ability; it exhibited the worst AUROC among our 11 models. Notably, any type of posterior scleral measure, including TEPS, may be a required condition to reach clinically useful competency for discrimination of pathologic myopic eyes.

This study was limited by its retrospective design. Although we validated the diagnostic performance of the model by means of training and validation sets, a prospective investigation of the data is necessary for confirmation. Second, our novel TEPS index does not reflect the detailed geometry of the posterior sclera; it only describes a portion of the sclera curvature. A more detailed geometry and wider range of scan data from an advanced device may provide superior predictability. Third, the data set was constructed using information collected from patients referred to tertiary ophthalmology institutes, in whom the prevalence of pathologic myopia is high; thus, the results may not be representative of general populations who exhibit lower prevalences of pathologic myopia. Fourth, the patients were mostly of Korean ethnicity; the accuracy of this model, when applied to individuals of other ethnic groups, remains unclear. The validity of this model must be confirmed in community populations of individuals with various ethnicities. Fifth, our study population had a class imbalance of 728 in the normal group and 132 in the patient group. The data imbalance was alleviated by the SMOTE method; however, sensitivity remained limited throughout the analysis. To overcome this limitation, future studies should collect large amounts of data. Sixth, assessment of the number of coronal sections to establish anteroposterior depth is an estimation, rather than an accurate measurement. However, there remains no established method for measurement of the exact depth of the posterior globe in vivo. Our method is adequate for comparison of posterior globe elevations among patients when a consistent scanning protocol is used. Lastly, the positions of the fovea, optic disc, and DPE were consistent only when patients’ eyes were fixated on the scanning light. Nonetheless, all ocular imaging apparatuses assume that the patient maintains fixation on the scanning light throughout the scanning process. Therefore, as with other parameters assessed by ocular imaging (e.g., peripapillary atrophy, optic disc tilt, and optic disc torsion), the three key indicator positions are reproducible if proper fixation is achieved.

## Conclusion

Our SVM model provides a simple and accurate modality for identification of eyes with pathologic myopia. The diagnostic accuracy of the SVM classifier was limited when using only the conventional indices of AxL and SCT; however, superior accuracy was achieved when the defined tomographic parameters of posterior globe measurements were incorporated. This quantitative tool may help clinicians to detect eyes with pathologic myopia. Future studies and machine learning algorithm development will focus on validation of our model with respect to community-based populations and multi-ethnic groups.

## Supplementary Information

Supplementary Information 1.

Supplementary Figure legend

Supplementary Figure.
