# Supplementary material for: Machine learning prediction of pathologic myopia using tomographic elevation of the posterior sclera
Source: Sci Rep. 2021 Mar 26;11:6950. doi: 10.1038/s41598-021-85699-0 (PMC7997908; doi:10.1038/s41598-021-85699-0)
Supplement: Supplementary file 1 — Supplementary Information 1. [file 41598_2021_85699_MOESM1_ESM.docx]

**Machine Learning Prediction of Pathologic Myopia Using Tomographic Elevation of the Posterior Sclera**

Yong Chan Kim, M.D., Ph.D. ^1^, Dong Jin Chang, M.D., Ph.D. ^2, 3^, So Jin Park ^2^,

In Young Choi, Ph.D. ^2^, Ye Seul Gong, M.D. ^1^, Hyun-Ah Kim, M.D. ^1^, Hyung Bin Hwang, M.D., Ph.D. ^1^, Kyung In Jung, M.D., Ph.D. ^4^, Hae-young Lopilly Park, M.D., Ph.D. ^4^, Chan Kee Park, M.D., Ph.D. ^4^, , Kui Dong Kang, M.D., Ph.D. ^1^

Department of Ophthalmology, Incheon St. Mary’s Hospital, College of Medicine, The Catholic University of Korea, Seoul, Republic of Korea ^1^

Department of Medical Informatics, College of Medicine, The Catholic University of Korea, Seoul, Republic of Korea ^2^

Department of Ophthalmology, Yeouido St. Mary’s Hospital, College of Medicine, The Catholic University of Korea, Seoul, Republic of Korea ^3^

Department of Ophthalmology, Seoul St. Mary’s Hospital, College of Medicine, The Catholic University of Korea, Seoul, Republic of Korea ^4^

**Conflict of Interest Disclosures: none**

**Correspondence:** Kui Dong Kang, M.D., Ph.D.

Department of Ophthalmology, Incheon St. Mary’s Hospital, College of medicine, The Catholic University of Korea, Republic of Korea, 56, Dongsu-ro, Bupyeong-gu, Incheon, 21431, Republic of Korea

Tel.: 82-032-280-5110     FAX: 82-032-280-5118 E-mail: [cmceyebank@gmail.com](mailto:cmceyebank@gmail.com)

**Supplementary Table 1.** Tuning Parameters for gamma and cost value in RBF kernel.

|  | Gamma | Cost | Error | Dispersion |
| --- | --- | --- | --- | --- |
| 1 | 0.01 | 0.01 | 0.474227 | 0.034019 |
| 2 | 0.1 | 0.01 | 0.149485 | 0.041594 |
| 3 | 1 | 0.01 | 0.474227 | 0.034019 |
| 4 | 10 | 0.01 | 0.474227 | 0.034019 |
| 5 | 0.01 | 0.1 | 0.168041 | 0.048611 |
| 6 | 0.1 | 0.1 | 0.112371 | 0.028524 |
| 7 | 1 | 0.1 | 0.113402 | 0.026618 |
| 8 | 10 | 0.1 | 0.474227 | 0.034019 |
| 9 | 0.01 | 1 | 0.128866 | 0.038955 |
| 10 | 0.1 | 1 | 0.113402 | 0.023307 |
| 11 | 1 | 1 | 0.089691 | 0.031514 |
| 12 | 10 | 1 | 0.110309 | 0.032255 |
| 13 | 0.01 | 10 | 0.125773 | 0.040602 |
| 14 | 0.1 | 10 | 0.101031 | 0.026971 |
| 15 | 1 | 10 | 0.068041 | 0.023907 |
| 16 | 10 | 10 | 0.106186 | 0.028772 |

Supplementary Table 2. Comparison of the model performance by each variables.

| No |  |  | **Actual Classes** | |  |  |  |  |
| --- | --- | --- | --- | --- | --- | --- | --- | --- |
|  | **Variables** | **Predicted** | Healthy | PM | **Accuracy** | **Sensitivity** | **Specificity** | **AUROC** |
| 1 | All Variables(gamma=1, Cost=10) | Healthy | 199 | 13 | 87.60% | 67.50% | 91.28% | 79.39% |
|  |  | PM | 19 | 27 |  |  |  |  |
| 2 | All Variables(gamma=1, Cost=1) | Healthy | 197 | 7 | 89.15% | 82.50% | 90.37% | 86.43% |
|  |  | PM | 21 | 33 |  |  |  |  |
| 3-1 | All Variables(gamma=1/data dimension, Cost=1) | Healthy | 204 | 8 | 91.47 % | 80.00% | 93.58% | 86.79% |
|  |  | PM | 14 | 32 |  |  |  |  |
