# Supplementary material for: Machine learning prediction of pathologic myopia using tomographic elevation of the posterior sclera
Source: Sci Rep. 2021 Mar 26;11:6950. doi: 10.1038/s41598-021-85699-0 (PMC7997908; doi:10.1038/s41598-021-85699-0)
Supplement: Supplementary file 2 — Supplementary Figure legend [file 41598_2021_85699_MOESM2_ESM.docx]

**Supplementary Figure1.** Bland-Altman plots of the four TEPS index. The upper and lower dotted red lines are designated as the 95 percent confidence intervals and the middle dotted line as the mean. The red dots are the value from the healthy myopia group (group A) and the blue dots are the value from the pathologic myopia group (group B).
