# Supplementary figures and images for: Machine learning prediction of pathologic myopia using tomographic elevation of the posterior sclera
Source: Sci Rep. 2021 Mar 26;11:6950. doi: 10.1038/s41598-021-85699-0 (PMC7997908; doi:10.1038/s41598-021-85699-0)

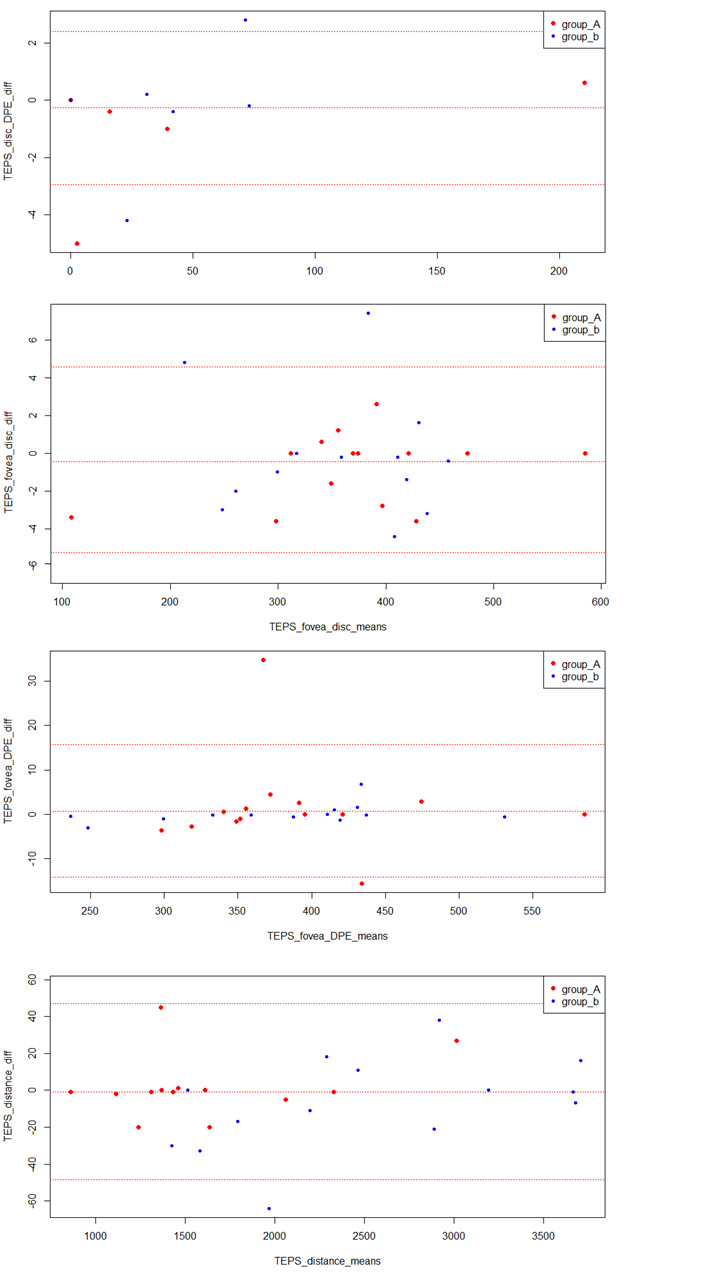

Supplement: Supplementary file 3 — Supplementary Figure. [file 41598_2021_85699_MOESM3_ESM.png]
